# Postural Instability Induced by Visual Motion Stimuli in Patients With Vestibular Migraine

**DOI:** 10.3389/fneur.2018.00433

**Published:** 2018-06-07

**Authors:** Yong-Hyun Lim, Ji-Soo Kim, Ho-Won Lee, Sung-Hee Kim

**Affiliations:** ^1^Department of Neurology, Kyungpook National University School of Medicine, Kyungpook National University Chilgok Hospital, Daegu, South Korea; ^2^Department of Neurology, Seoul National University College of Medicine, Seoul National University Bundang Hospital, Seoul, South Korea

**Keywords:** migraine, vertigo, posture, balance, motion sickness

## Abstract

Patients with vestibular migraine are susceptible to motion sickness. This study aimed to determine whether the severity of posture instability is related to the susceptibility to motion sickness. We used a visual motion paradigm with two conditions of the stimulated retinal field and the head posture to quantify postural stability while maintaining a static stance in 18 patients with vestibular migraine and in 13 age-matched healthy subjects. Three parameters of postural stability showed differences between VM patients and controls: RMS velocity (0.34 ± 0.02 cm/s vs. 0.28 ± 0.02 cm/s), RMS acceleration (8.94 ± 0.74 cm/s^2^ vs. 6.69 ± 0.87 cm/s^2^), and sway area (1.77 ± 0.22 cm^2^ vs. 1.04 ± 0.25 cm^2^). Patients with vestibular migraine showed marked postural instability of the head and neck when visual stimuli were presented in the retinal periphery. The pseudo-Coriolis effect induced by head roll tilt was not responsible for the main differences in postural instability between patients and controls. Patients with vestibular migraine showed a higher visual dependency and low stability of the postural control system when maintaining quiet standing, which may be related to susceptibility to motion sickness.

## Introduction

Vestibular migraine (VM) is one of the most common disorders causing dizziness/vertigo (1). Patients with VM are more vulnerable to motion sickness, and motion sensitivity with bouts of motion sickness occur in two-thirds of patients with migraine (2). This susceptibility extends beyond motion-based stimuli to visual stimuli that create an illusion of movement. The mechanisms underlying vestibular symptoms in migraine—including episodic dizziness and enhanced susceptibility to motion sickness—remain to be clarified (3).

The most widely accepted explanation of motion sickness is the sensory conflict theory (4), which states that a signal combination that violates habitually experienced patterns and thus causes a mismatch between the expected and the perceived senses results in motion sickness (5). This theory explains visually induced motion sickness in the context that the illusory sensation of self-movements when watching moving visual stimuli, called vection, does not match vestibular or proprioceptive cues indicating that the body is still. In contrast, the subjective vertical theory states that motion sickness is induced by a condition in which the perceived vertical—as determined on the basis of integrated information from the visual, vestibular, and proprioceptive systems—is not accordant with the expected vertical as predicted on the basis of previous experiences (6). Although the conflict in the perception of verticality is emphasized in the subjective vertical theory, this appears to parallel the sensory conflict theory (4). It has also been hypothesized that motion sickness occurs from instability in the control of the posture of the body or its segments (7). This hypothesis proposes that motion sickness occurs when combined motion and visual stimuli actually disturb postural stability, defined as the state in which uncontrolled movements of the kinetic system are minimized (7, 8). This postural instability theory focuses on the mechanical aspect of an individual interaction between the subject and the environment rather than on innate vestibular neurophysiology, and can be supported by evidence being obtainable from motion outputs. The postural instability theory states that postural instability precedes the onset of motion sickness (9, 10).

Previous studies on motion sickness in patients with migraine have found underlying vestibular disturbances or abnormalities in eye movements, which have usually been explained based on the sensory conflict theory together with vestibulocerebellar pathology (11–13). Patients with VM frequently show spontaneous or positional nystagmus (14), inaccurate saccades (13), an increased time constant for the vestibulo-ocular reflex (VOR), and greater suppression of the postrotatory nystagmus with forward head tilt (15). Compared with controls, VM patients show a weaker correlation between dumping of the VOR and shifting of its rotational axis during postrotational tilts (16), which suggests dysfunction of central processing for resolving intravestibular sensory conflicts between cues from the otolith and semicircular canals. Patients with VM and vestibular abnormalities also show poor performance in static posturography and seem to rely more on visual cues for balance control (17). However, there has been no quantitative analysis of posture when patients with migraine are exposed to a situation that can generate motion sickness.

In this study we measured postural instability in controls and VM patients when they were exposed to a moving visual display. We hypothesized that the severity of posture instability induced by the applied visual motion stimuli while maintaining a static stance are related to the susceptibility to motion sickness. The investigation also imposed variations in the stimulated retinal field and the head posture in order to determine the factors that increase postural instability.

## Materials and methods

### Participants

This study enrolled 18 patients with VM between October and December 2016 who met the diagnostic criteria of definite VM produced by a working group of the Barany Society (18). The study population comprised 16 women and 2 men with a mean age of 45.67 years (standard deviation of 12.55 years). Thirteen healthy volunteers comprising 8 women and 5 men with a mean age of 37.62 years (standard deviation of 15.31 years) served as the age matched controls. Patients were excluded if they had impaired attention or a history of other neurologic, vestibular, visual, or spinal disorders. The video nystagmography and head impulse tests were applied to the patients with VM, and those with abnormalities of the vestibular function tests suggestive of the other vestibular disorder were excluded in this study. All of the participants had normal or corrected-to-normal vision. The patients were investigated when they were not experiencing headache or dizziness/vertigo.

### Apparatus

Postural stability was measured with a six-degrees-of-freedom system (G4^TM^, Polhemus, Colchester, USA). The operation of the G4™ system is based on an electromagnetic field being detected by three sensors attached to the vertex and the seventh cervical and fifth lumbar vertebrae. The amplitude of the electromagnetic field at the sensors and their orientation in it are detected at 120 Hz and transmitted wirelessly to a hub, which yields real-time 3D motion data. The sensing axes are oriented along the anatomical medial–lateral (*x*-axis), anterior–posterior (*y*-axis), and vertical (*z*-axis) directions. Visual motion stimuli were programmed in Python using PsychoPy2 software on a PC and presented on a 55-inch monitor (LN55C632, Samsung, Korea) positioned 90 cm from the participant's cornea. The height of the monitor was adjusted so that the stimuli were presented at the same height as the participant's eyes.

### Experimental protocol

**Figure 1:**
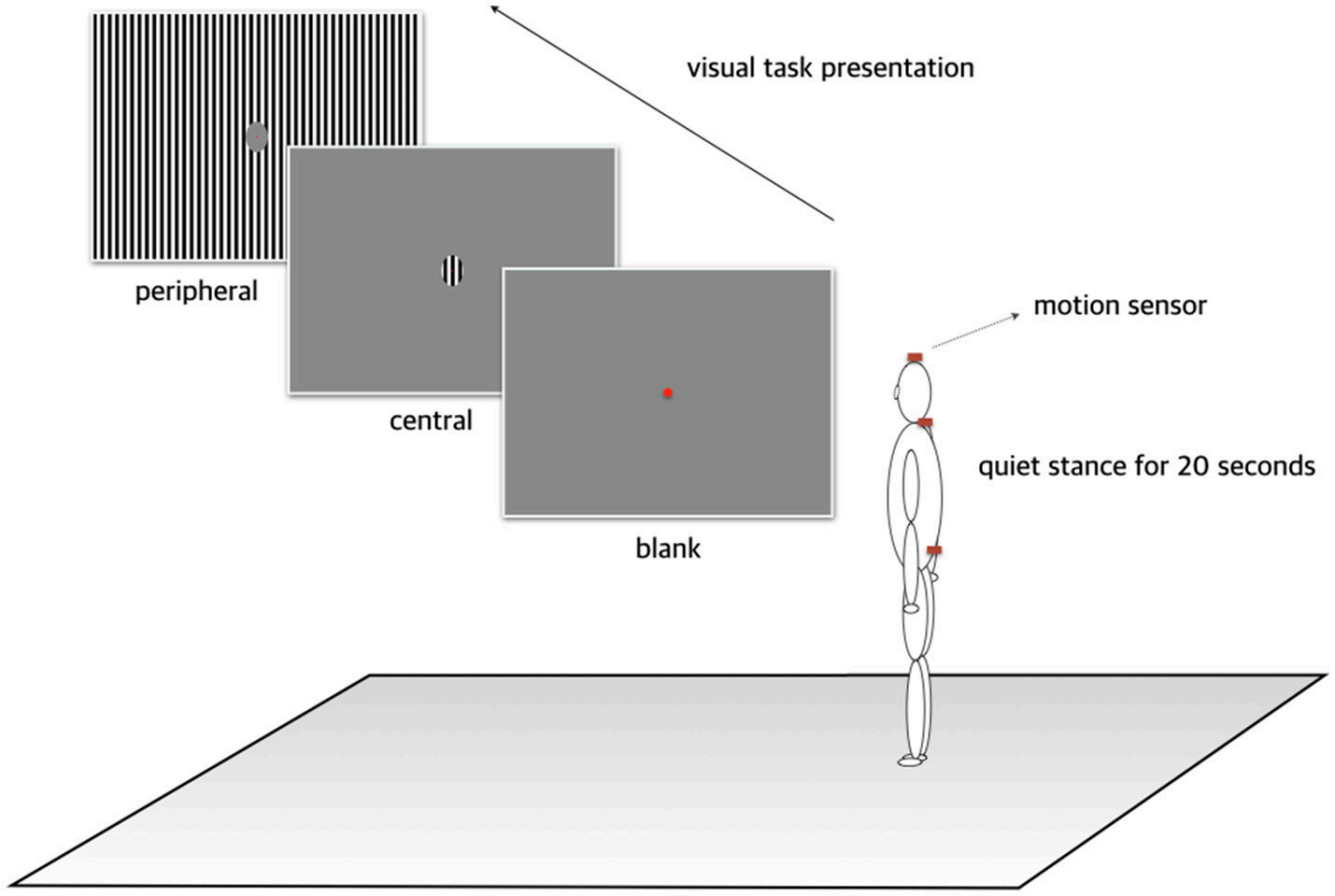
Experimental protocol. Each participant was instructed to stand with heels flat on the floor and view a red dot presented at the center of the monitor. Three visual stimuli were then projected on the display: (1) a gray background, (2) a visual motion stimulus comprising a black-and-white grating within a central circle subtending a central visual angle of approximately 5.0° moved at a spatial frequency of 7/200 cycles per degree of visual angle, and (3) the grating visual motion stimuli filling the visual periphery but with the central circle filled with gray.

Each participant was instructed to stand with heels flat on the floor and view a red dot presented at the center of the monitor. Three visual stimuli were then projected onto the display: (1) *blank*, comprising a gray background, (2) *central*, comprising a visual motion stimulus of a rightward-moving black-and-white grating within a central circle with a diameter of 7.85 cm, and (3) *peripheral*, comprising the grating visual motion stimulus filling the periphery but with the central circle filled with gray (Figure 1). The central circle subtended a visual angle of approximately 5.0°. The moving visual stimuli were displayed with a spatial frequency of 7/200 cycles per degree of visual angle. Each trial involving three visual stimuli was performed under two posture conditions: (1) head upright and (2) head tilted 30° to one side in the roll plane. Each participant therefore performed six trials (three visual × two posture conditions), and the duration of each trial was restricted to 30 s in order to minimize fatigue associated with a prolonged stance and adaption to visual stimuli. Participants were asked to close their eyes and rest for at least 30 s between trials. During a resting period, the severity of dizziness and/or nausea induced by each trial was measured using a subjective 10-point motion-sickness scale ranging from none to severe.

### Data analysis

During the experimental sessions, the raw signals were sampled synchronously at 120 Hz and stored in a host PC via the hub device. The time series of the sensor trajectories in the three directions were low-pass filtered at 10 Hz using a fourth-order Butterworth filter to remove noise and tremor signals. After preprocessing of the signals, the measured data consisted of the dynamic velocity and acceleration while maintaining a quiet stance. Root mean square (RMS) values were calculated based on the summated magnitude of the motion in three axes (19) as follows:

$$\begin{array}{l} summated\;velocity=\sqrt{v_{x}^{2}+v_{y}^{2}+v_{z}^{2}} \end{array}$$

$$\begin{array}{l} summated\;accelaration=\sqrt{a_{x}^{2}+a_{y}^{2}+a_{z}^{2}} \end{array}$$

where $v_{x}^{2}$, $v_{y}^{2}$, and $v_{z}^{2}$ are the filtered velocities along the *x*-, *y*-, and *z*-axes, respectively, and $a_{x}^{2}$, $a_{y}^{2}$, and $a_{z}^{2}$ are the corresponding filtered accelerations.

Postural stability was also analyzed using the sway area calculated as the area covering the trajectory formed by both the *x*- and *y*-axes using the convex hull algorithm (20).

### Statistics

SPSS software (version 20.0, SPSS, Chicago, USA) was used to apply repeated-measures analysis of variance (ANOVA) with one between-subjects factor (participant groups) and three within-subject factors (body segments, visual stimuli, and head postures). We employed simple main-effects testing to identify pairwise differences in any significant interaction. The cutoff for statistical significance was a probability value of *p* < 0.05.

## Results

### RMS velocity

**Figure 2:**
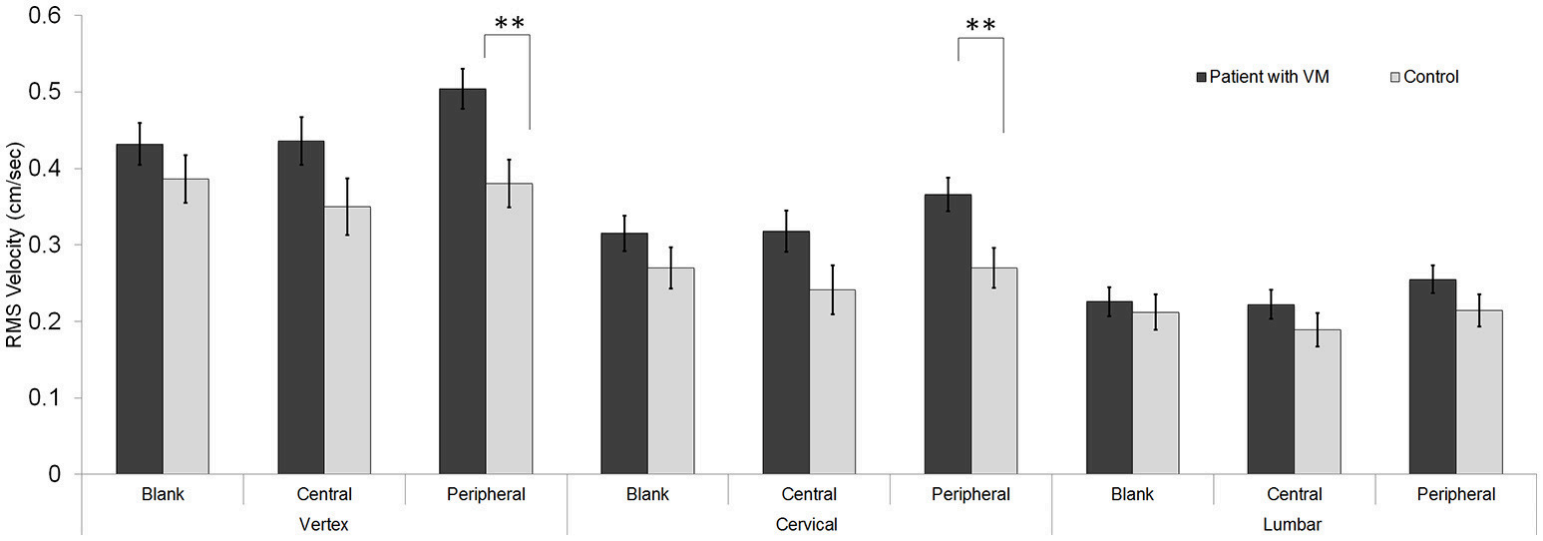
Velocity. Compared with the controls, patients with vestibular migraine (VM) showed a significantly higher root mean square (RMS) velocity during a static stance under two conditions: the peripheral visual stimuli combined with the vertex, and the peripheral visual stimuli combined with the cervical area [^**^p < 0.01 in three-way analysis of variance (ANOVA) for multiple comparisons]. Data are mean and standard-error values.

Repeated-measures ANOVA of the RMS velocity identified a significant three-way interaction for body segments × visual stimuli × participant groups [*F*_(4, 128)_ = 2.97, *p* < 0.05]. The RMS velocity of the patients [0.34 ± 0.02 cm/s (mean ± standard error)] was higher than that of the controls (0.28 ± 0.02 cm/s). The simple main-effects testing of the three-way interaction revealed that the intergroup difference in RMS velocity occurred under two conditions: peripheral visual stimuli in combination with the vertex (mean difference [*Δ*] = 0.13 ± 0.04, *F*_(1, 29)_ = 9.5, *p* < 0.01), and peripheral visual stimuli in combination with the cervical spine [*Δ* = 0.10 ± 0.03, *F*_(1, 29)_ = 8.13, *p* < 0.01; Figure 2].

### RMS acceleration

**Figure 3:**
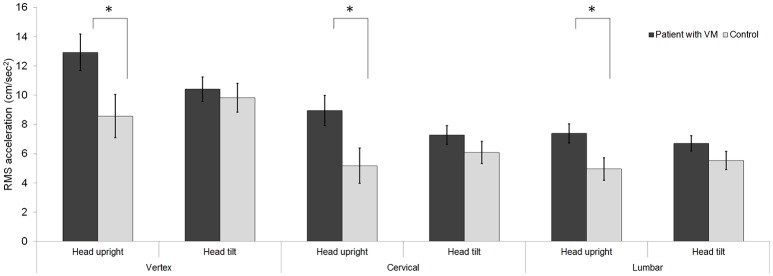
Acceleration. A head-upright posture in combination with each body segment resulted in the RMS acceleration during a static stance differing significantly between the control and patient groups (^*^p < 0.05 in three-way ANOVA for multiple comparisons). In contrast, a head-tilted posture resulted in no significant intergroup difference in the acceleration. Data are mean and standard-error values. VM, vestibular migraine.

Repeated-measures ANOVA of the RMS acceleration identified a significant three-way interaction for body segments × head posture × participant groups [*F*_(2, 58)_ = 5.66, *p* < 0.01]. The RMS acceleration of the patients (8.94 ± 0.74 cm/s^2^) was higher than that of the controls (6.69 ± 0.87 cm/s^2^). The simple main-effects testing of the three-way interaction showed that three conditions resulted in an intergroup difference in RMS acceleration: a head-upright posture in combination with the vertex [*Δ* = 4.36 ± 1.93, *F*_(1, 29)_ = 5.11, *p* < 0.05], a head-upright posture in combination with the cervical spine [*Δ* = 3.78 ± 1.58, *F*_(1, 29)_ = 5.71, *p* < 0.05], and a head-upright posture in combination with the lumbar spine [*Δ* = 2.43 ± 1.01, *F*_(1, 29)_ = 5.77, *p* < 0.05; Figure 3]. A head-tilted posture in combination with each body segment did not result in any significant intergroup difference in the RMS acceleration.

### Sway area

**Figure 4:**
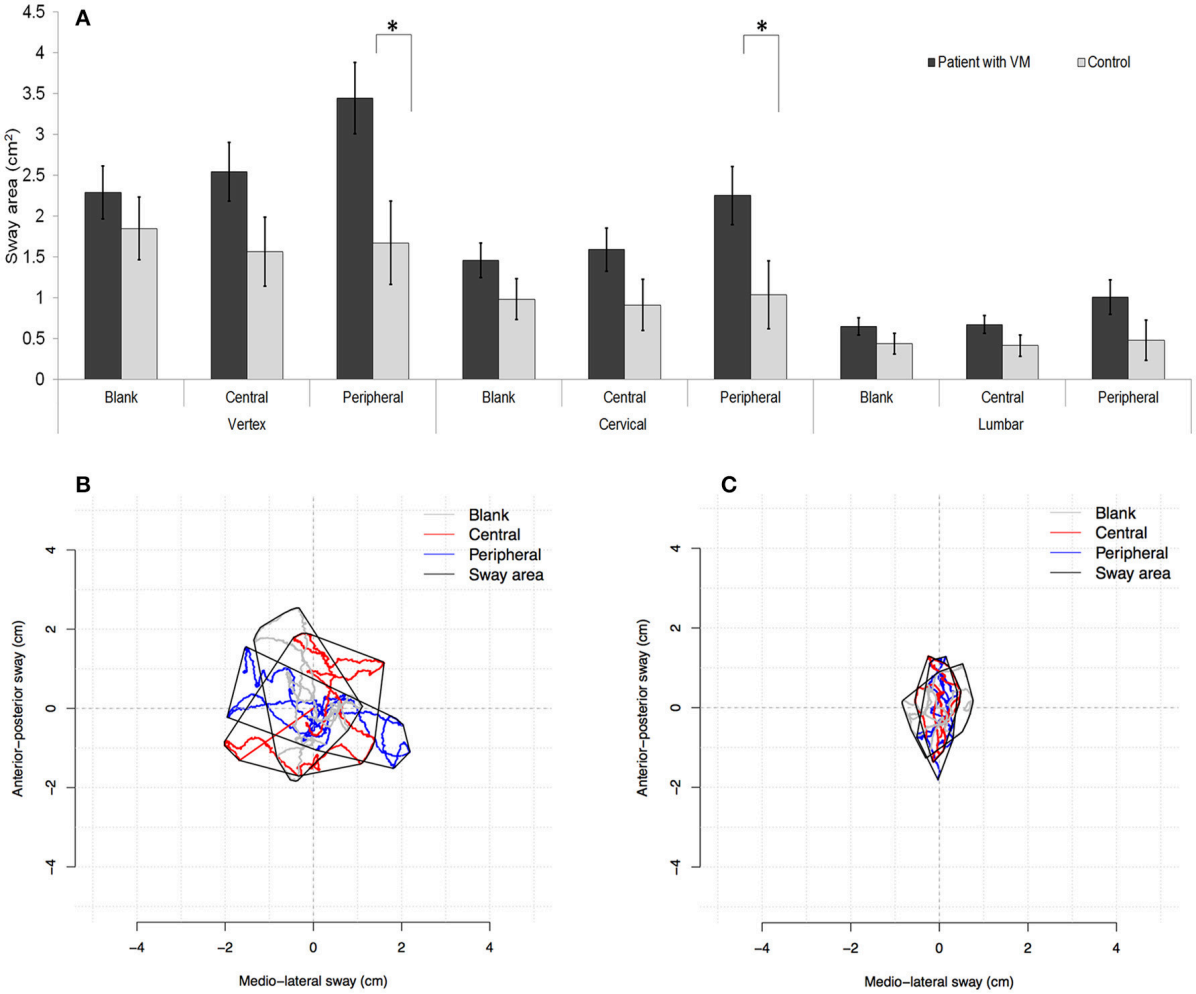
Sway area. Compared with the controls, patients with vestibular migraine (VM) exhibited a significantly larger sway area when maintaining a static stance under the following conditions: peripheral stimuli combined with the vertex, and peripheral stimuli combined with the cervical spine **(A)**. (^*^p < 0.05 in three-way ANOVA for multiple comparisons.) Examples of the sway area that covered the trajectory formed by both the medial–lateral and anterior–posterior axes for a patient with VM **(B)** and a control **(C)**.

Analysis of the sway area also revealed a significant three-way interaction for body segments × visual stimuli × participant groups [*F*_(4, 116)_ = 2.66, *p* < 0.05]. The sway area of the patients (1.77 ± 0.22 cm^2^) was significantly larger than that of the control (1.04 ± 0.25 cm^2^). The simple main-effects testing of the three-way interaction showed that the intergroup difference occurred under two conditions: peripheral visual stimuli in combination with the vertex [*Δ* = 1.77 ± 0.67, *F*_(1, 29)_ = 7.01, *p* < 0.05], and peripheral visual stimuli in combination with the cervical spine [*Δ* = 1.22 ± 0.55, *F*_(1, 29)_ = 4.94, *p* < 0.05; Figure 4]. Each visual condition in combination with the lumbar spine did not result in any intergroup difference in the sway area.

### Subjective sickness scale

**Figure 5:**
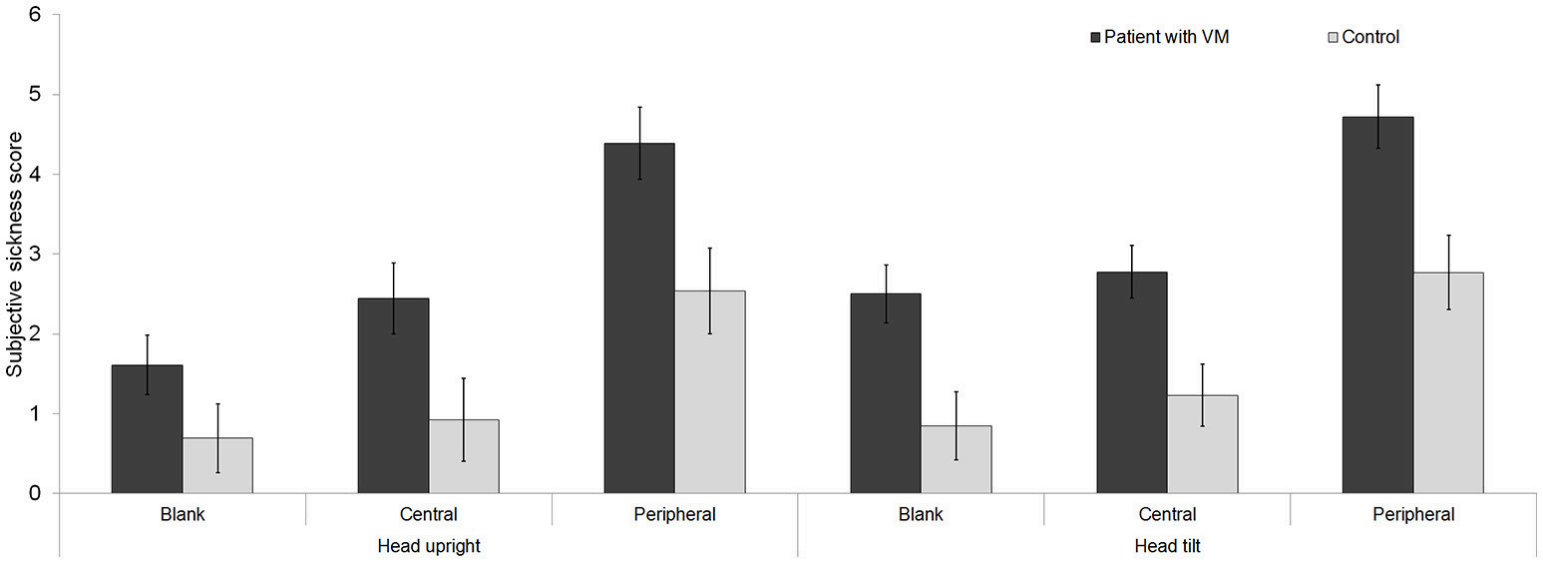
Subjective sickness scale. The visual motion stimuli increased the scores on the subjective sickness scale in both patients with vestibular migraine (VM) and controls, especially when the stimuli were presented in the peripheral field. Relative to a head-upright posture, a head-tilt posture in combination with each visual stimulus increased the scores on the subjective sickness scale in both patients and controls, but there was no intergroup difference in the magnitude of the score change.

The scores on the subjective sickness scale (SSS) differed significantly between the patient and control groups [*F*_(1, 29)_ = 11.08, *p* < 0.01] and also according to the visual stimuli [*F*_(2, 58)_ = 47.03, *p* < 0.001]. In both the patients and controls, visual motion stimuli increased the scores on the subjective sickness scale, especially when the stimuli were presented in the peripheral field (blank < central < peripheral) (Figure 5). Compared with the head-upright posture, a head-tilt posture in combination with each visual condition increased the scores on the SSS in both groups, but there was no intergroup difference in the magnitude of the score change [*F*_(1, 29)_ = 3.00, *p* = 0.09].

## Discussion

This study evaluated postural stability in VM patients and controls using a visual motion paradigm with two conditions of the stimulated retinal field and the head posture. We found that three aspects of postural stability differed between VM patients and controls: RMS velocity, RMS acceleration, and sway area.

The results for RMS velocity indicated that patients with VM were more affected by visual motion stimuli and exhibited more movements when attempting to maintain a static stance, whereas the controls could maintain unperturbed standing despite changes in visual stimuli. Patients with VM generated larger motions of the head and neck during quiet standing when visual motion stimuli were presented in the retinal periphery. This is consistent with previous findings of the visual perception of movements being a determinant factor in maintaining the equilibrium, in which the peripheral vision plays the main role (21, 22). The visual stimuli arising from the retinal periphery are transmitted to the vestibulocerebellum (23) that is known to show dysfunction in migraineurs. Interictal neurotologic evaluation in patients with migraine showed oculomotor abnormalities which are mostly attributed to vestibullocebellar dysfunction (13). The vestibulocerebellum including the nodulus/uvula is responsible for controls of the vestibular storage mechanism that takes an important role for minimizing intra-vestibular sensory conflict (24). Vestibular migraineurs indeed showed impaired ability of the central integration of rotational cues from the semicircular canals and gravitational cues from the otolith organs (16). In addition to dysfunction of the subcortical circuits, the cortical pathway connected to the retinal periphery responsible for visual motion processing may also play a role for enhanced motion sickness in vestibular migraineurs. The previous study using ^18^F-fluorodeoxy glucose positron-emission tomography study showed increased metabolism in the temporo-parieto-insular areas and bilateral thalami, which indicates activation of the vestibulo-thalamo-cortical pathway during the attacks of vestibular migraine (25).

Relative to the controls, our VM patients exhibited higher accelerations of every evaluated body segment while maintaining a static stance with a head-upright posture. In contrast, the RMS acceleration in static stance with the head tilted did not differ between the patients and controls. The Coriolis effect refers to the condition in which the subject is rotating around an earth vertical axis and the head is tilted around another axis (26). In that situation an illusory tilt sensation is usually perceived with strong feelings of sickness. The pseudo-Coriolis effect refers to a similar condition where the illusory sense of rotation arises from moving visual stimuli, and rolling the head out of the axis of visual rotation elicits a tilting sensation and vegetative symptoms (27). Both the Coriolis and pseudo-Coriolis effects can be explained by the subjective visual vertical theory (26). Additional head rolling during an ongoing illusory rotatory sensation leads to shifting of the perceived vertical vector, which is in conflict with the expected earth verticality. In the present study, the pseudo-Coriolis effect indeed produced strong motion sickness in both patient and control controls. However, the scores on the SSS increased similarly in the two groups during the pseudo-Coriolis condition, and the pseudo-Coriolis effect did not produce an intergroup difference in RMS acceleration. Together these findings suggest that, besides conflicts between the perceived and expected verticality, the additional factor(s) such as different visual dependency is required for explaining the enhanced motion sickness in our patients with VM.

The sway area for VM patients was larger when the moving visual stimuli were presented to the retinal periphery, and these patients had higher scores on the SSS when visual stimuli moved in the peripheral area. These observations suggest that patients with VM are more dependent than controls on visual inputs for maintaining a static stance. A static stance is not a kind of resting state lacking visible activities, but instead is an active state maintained by the posture control system that cooperates with the multidimensional sensory system. Motion observed in both the patient and control groups should not be regarded as noise during passive unperturbed standing. Rather, each parameter of acceleration, velocity, and calculated sway area reflects incessant activation of the body segments required to maintain the voluntary standing posture. Given that the activation while holding the same posture was greater in VM patients than in controls, the posture control system in these patients seems to have relatively low effectiveness. In addition to dysfunction of the vestibulocerebellum, these characteristics of higher dependency on visual inputs and lower effectiveness of the kinetic system may consequently lead to vulnerability to disequilibrium in patients with VM, which also contribute to the symptomatology of VM that includes motion sickness, nausea, and dizziness.

Stability of the truncal posture during standing can be quantitative indicators of impaired balance ability in neurologic disorders (28). Given that cervical proprioception is one of the important intra-conflictive sensory components causing motion sickness, postural instability that precedes motion sickness may not only be confined to the body center, but the other segments such as the head and neck may show rather more movements for maintaining a stance in patients with VM. However, the traditional posturography allows for measuring the 2D movements in transversal plane and produces parameters associated with the center of pressure movements of the whole body (29). Instead of the conventional platforms, assessment of body movements by the triaxial sensor system can yield high-accuracy measurement of the 3D motions of the multiple body segments (29). Analyses of the sway area in our study indeed revealed that patients with VM showed larger motions of the head and neck, which are more distant from the center of body pressure than the lumbar region. Since the triaxial real-time sensor system used in this study yielded extensive data requiring complex pre-processing, however, our study had limits of the participants number.

Since we adopted a visual motion-based paradigm for motion sickness rather than a paradigm based on physical motion, there was underlying predominance of vision in this study. Vision is the main contributor to the sensation of self-motion when experiencing or viewing rotation at a constant velocity, whereas the labyrinthine organs—whose specific stimulus is acceleration—can sense changes in velocity induced by head movements. We thus employed a head-tilted posture as an independent variation factor for eliciting the pseudo-Coriolis effect, which is known to represent strong nauseogenic stimulation. Given that the pseudo-Coriolis effect was not responsible for the main differences in postural instability and the scores on the SSS between our patients and controls, the dominancy of vision appears to be an important factor for increased postural instability and the increased experience of motion sickness in patients with VM.

## Conclusion

This study quantified postural stability by measuring the motions of three body segments while standing participants were exposed to visual motion stimuli. Postural stability differed significantly between the controls and VM patients when tested with a paradigm that generated linear vection and the pseudo-Coriolis effect. In contrast to the controls, patients with VM showed marked postural instability of the head and neck when the visual stimuli were presented in the retinal periphery. Patients with VM showed a higher visual dependency and the low stability of the postural control system when maintaining quiet standing, which may be related to their susceptibility to motion sickness.

## Ethics statement

This study was carried out in accordance with the recommendations of the Institutional Review Board of Kyungpook National University Chilgok Hospital. The protocol was approved by the Institutional Review Board of Kyungpook National University Chilgok Hospital. All subjects gave written informed consent in accordance with the Declaration of Helsinki.

## Author contributions

Y-HL conducted the experiments, analyzed the data, and wrote the manuscript. J-SK contributed to critical revision of the manuscript. H-WL contributed to conduction of the experiments and collection of the data. S-HK designed the study, analyzed and interpreted the data, and wrote the manuscript.

### Conflict of interest statement

The authors declare that the research was conducted in the absence of any commercial or financial relationships that could be construed as a potential conflict of interest.
